# Gossypol Affects Viral Replication by Inhibiting Pseudorabies Virus Adsorption

**DOI:** 10.1155/2023/9073566

**Published:** 2023-11-11

**Authors:** Yilin Bai, Bingqian Su, Xuan Cheng, Qianwen Liu, Lei Zeng, Pengfei Fu

**Affiliations:** ^1^Laboratory of Indigenous Cattle Germplasm Innovation, School of Agricultural Sciences, Zhengzhou University, Zhengzhou 450046, Henan, China; ^2^Zhengzhou Research Base, State Key Laboratory of Cotton Biology, School of Agricultural Sciences, Zhengzhou University, Zhengzhou 450046, Henan, China; ^3^College of Veterinary Medicine, Henan Agricultural University, Zhengzhou 450046, Henan, China; ^4^College of Life Science and Engineering, Henan University of Urban Construction, Pingdingshan 467044, Henan, China

## Abstract

Pseudorabies virus (PRV) has spread widely in swine herds since it was introduced into China, and PRV variants have appeared in many regions in China since 2011. Owing to the ongoing emergence of viral mutations, the protective efficacy of PRV vaccines faces great challenges. Gossypol is a polyphenolic natural compound purified from cotton roots, stems, or seeds. This study found that gossypol has a notable inhibitory effect on PRV. Based on the biological effects of gossypol, the anti-PRV effect of gossypol was studied from the aspects of mitochondrial damage, reactive oxygen species, energy metabolism, and autophagy. The results showed that gossypol had no direct inactivation effect on PRV in solution within concentrations of ≤3 *µ*M; gossypol did not stimulate interferon-*β* to exert an anti-PRV effect by triggering mitochondrial DNA leakage; the anti-PRV effect of gossypol was reactive oxygen species and ATP-independent in cells; and the PRV-inhibitory effect of gossypol was independent of its induced autophagy. By adding gossypol to PRV-infected cells at different stages of infection and performing virus titer detection, atomic force microscopy, qPCR, fluorescence microscopy, and firefly luciferase activity assays, we found that gossypol exerted an anti-PRV effect by inhibiting the adsorption of PRV on the cell surface.

## 1. Introduction

Pseudorabies virus (PRV) is an enveloped, double-stranded, linear DNA virus with a genome length of approximately 150 kb, which belongs to the family *Herpesviridae*, subfamily *Alphaherpesvirus*, and is a member of the genus *Varicella* [1]. PRV has been very harmful for swine herds, especially since 2011. Some pig farms in China that had immunized their herds with traditional commercial PRV vaccines have experienced a new round of PRV infection, caused by PRV mutants, and these mutant viruses are severely affecting the pig industry in China [2–4]. At present, the prevention and control of PRV in pig farms relies mainly on vaccination. However, the protective efficacy of PRV vaccine is being challenged by the continuous mutation of PRV. Therefore, it is of great importance to find effective anti-PRV drugs and explore their mechanism of action.

Gossypol is a yellow fat-soluble polyphenolic compound extracted from the seeds, roots, or stems of cotton or other Malvaceae plants [5, 6]. Initially, gossypol was known for its contraceptive properties [7, 8]; an improved understanding of gossypol has led to this compound or its derivatives being used to treat female hormone-dependent diseases, such as female uterine fibroids, dysfunctional uterine bleeding, and endometriosis [9]. Recent studies have shown that gossypol and its derivatives have a variety of therapeutically useful biological properties, such as being anti-inflammatory [10], anti-parasitic, antiviral, and antioxidant [11–14]. In the present study, we found that gossypol has a significant inhibitory effect on PRV. It is known that gossypol can produce an antitumor effect by inducing mitochondrial dysfunction, autophagy, or apoptosis through specific pathways that vary by the type of tumor cell [15, 16]. Mitochondria are an important organelle in eukaryotic cells, which are not only the energy factories and key metabolic hubs of cells [17], but also exert important functions regarding innate immune responses [18]. When mitochondrial damage is induced by some external or internal factors, the leakage of mitochondrial DNA (mtDNA) into the cytoplasm can activate the cGAS–STING signaling pathway and induce the production of antiviral factors such as interferon 1 (IFN-1) [19]. Therefore, given the known mechanisms for other cellular effects induced by gossypol, we investigated mitochondrial damage, reactive oxygen species (ROS), energy metabolism, and autophagy to determine the anti-PRV effect of gossypol.

## 2. Materials and Methods

### 2.1. Cells, Strains, and Plasmids

Pig kidney epithelial cells (PK-15), mouse embryonic fibroblasts (NIH-3T3), and human embryonic kidney cells 293 (HEK293) were cultured in DMEM (Gibco) supplemented with 10% FBS (Gibco) at 37°C in an atmosphere with 5% CO_2_. PRV variant HN1201 (GenBank Accession Number: KP722022) was kindly donated by Professor Ke-Gong Tian from the School of Veterinary Medicine of Henan Agricultural University; rPRV HN1201-EGFP-Luc recombinant PRV was constructed in the previous studies [20]. Plasmids mCherry-Mito-7 (#55102), pGFP-LC3-RFP (#84573), psPAX2 (#12260), and pMD2.G (#12259) plasmids were purchased from Addgene (MA, USA). PRV gH standard plasmids and pLVX-IRES-Puro-RFP-GFP-fis1 plasmids were constructed by our laboratory.

### 2.2. Reagents and Antibodies

Rapamycin (HY-10219) and Gossypol (HY-13407) were purchased from MCE (New Jersey, USA). The Enhanced Mitochondrial Membrane Potential Assay kit with JC-1 (C2003S), Reactive Oxygen Species Assay kit (S0033S), and Enhanced ATP Assay kit (S0027) were purchased from Beyotime Biological Co., Ltd., and an animal tissue genomic DNA mini-extraction kit (DK611-02) was purchased from Shanghai Laifeng Biotechnology Co., Ltd. Anti-*β*-actin antibody (66009-1-l g) was purchased from Proteintech, and anti-PRV gE protein monoclonal antibody was prepared and stored by our laboratory. The antibodies anti-LC3 (4599) and anti-P62 (5114) were obtained from Cell Signaling Technology, and horseradish peroxidase- (HRP-) labeled goat anti-mouse IgG or goat anti-rabbit IgG antibodies were purchased from Beijing Dingguo Changsheng.

### 2.3. Cell Viability Assay

The cells were grown in a 96-well plate seeded with 6 × 10^3^ cells per well and then treated with complete cell culture medium containing gossypol at the specified concentration. The viability of cells after gossypol treatment was detected by applying the CCK-8 method according to instructions.

### 2.4. Detection of Viral Proliferation by Fluorescence Microscopy or Flow Cytometry

PK-15 cells were treated with Gossypol or DMSO at the specified concentrations and then inoculated with rPRV HN1201-EGFP-Luc at MOI = 0.01. These cells were observed and photographed with an inverted fluorescence microscope at an appropriate time after virus infection. For the detection of viral proliferation by flow cytometry, the cell culture medium was first discarded, and then the PK-15 cells were washed three times with 1 × PBS before an appropriate amount of 0.25% trypsin was added to digest the cells. After the cells were fully digested, they were centrifuged at 800 *g* for 5 min at room temperature; the resulting supernatant was discarded, and the pelleted cells were fully resuspended with an appropriate amount of PBS. The proportion of EGFP-positive cells in each sample was determined by using a Beckman CytoFLEX flow cytometer.

### 2.5. Western Blot

Whole-cell lysates were prepared with RIPA Lysis Buffer (P0013B, Beyotime) supplemented with protease inhibitors (4693116001, Roche). The protein concentration in the lysate was determined with a Bicinchoninic Acid (BCA) Protein Assay Kit (BCA01, DINGGUO Biotechnology). The lysates were then used in a western blot performed using conventional methods. Exposure imaging was performed with a GE Healthcare AI600 imaging system using Luminata Crescendo Western HRP Substrate (WBLUR0500, Millipore) chromogenic solution.

### 2.6. Detection of Cell Mitochondrial Morphology

PK-15 cells were inoculated into a 24-well plate (5 × 10^4^ cells/well) with prefabricated cell slides and then cultured at 37°C for 12 hr in an atmosphere with 5% CO_2_. The mitochondria-labeling plasmid mCherry-Mito-7 was transiently transfected into the cells using TurboFect™ Transfection Reagent (Thermo Fisher, R0533) in accordance with the manufacturer's protocol, and the cells were subsequently cultured for an additional 24 hr. After the cell culture medium was discarded, a complete cell culture medium containing gossypol at the indicated concentration was added, and the cells were cultured further. Cell slides were collected at specific times after the addition of gossypol, and the cells were fixed with 4% paraformaldehyde (PFA) solution for 30 min at 25°C. The cell fixative was then discarded, and the cells were washed three times with PBS, and their nuclei were stained with DAPI reagent for 10 min in the dark. The obtained cells were washed three times with PBS, washed three times with sterile ddH_2_O, and sealed, and the morphology of the mitochondria in these cells was observed and photographed under a Zeiss LSM710 confocal microscope.

### 2.7. Detection of Cell Mitochondrial Membrane Potential

PK-15 cells were seeded into 20-mm glass dishes at a density of 6 × 10^4^ cells/dish and then treated with gossypol at the indicated concentration for 24 hr. Cell mitochondria were stained using a Mitochondrial Membrane Potential Assay kit with JC-1 (C2006, Beyotime) in accordance with the manufacturer's instructions, then observed and photographed under a Zeiss LSM710 confocal microscope. The ratio of red to green fluorescence was analyzed with ImageJ software.

### 2.8. qRT-PCR

Total RNA was isolated with TRIzol Reagent (TaKaRa, 9108), and cDNA synthesis was performed with a PrimeScript™ RT Reagent kit (TaKaRa, RR047A) in accordance with the manufacturer's instructions. Data were normalized to the *β*-actin expression level for each sample by repeatedly performing qRT-PCR on three replicates with SYBR Premix Ex Taq (RR820A, TaKaRa), following the manufacturer's protocol. The relative expression change was calculated by the 2^−*ΔΔ*Ct^ method. Quantification of the PRV genome copies was performed as described previously [21]. The following primers were used for qRT-PCR: Porcine actin-F: 5′-CTGAACCCCAAAGCCAACCGT-3′; Porcine actin-R: 5′-TTCTCCTTGATGTCCCGCACG-3′; Porcine IFN-*β*-F: 5′-AGTTGCCTGGGACTCCTCAA-3′; Porcine IFN-*β*-R: 5′-CCTCAGGGACCTCAAAGTTCAT-3′; Porcine-mtDNA-F: 5′-GTACATAGCACATATCATGTC-3′; Porcine-mtDNA-R: 5′-TAAGGGGAAAGAGTGGGCGAT-3′; q-PRV gH-F: 5′-CTCGCCCTCGTCAGCAA-3′; and q-PRV gH-R: 5′-GCTGCTCCTCCATGTCCTT-3′.

### 2.9. ROS Detection

After PK-15 cells were treated with different concentrations of gossypol for 24 hr, the ROS in the cells were detected with a Reactive Oxygen Species Assay kit (S0033S, Beyotime) in accordance with the instructions, and the cells in each sample were finally observed and photographed under a Zeiss LSM710 confocal microscope.

### 2.10. Lentiviral Packaging of the pLVX-IRES-Puro-RFP-GFP-Fis1 Vector

HEK293T cells were spread into a T25 culture flask (1.5 × 10^6^ cells/flask) and then cultured for approximately 24 hr. When the level of cell confluency reached approximately 40%, 2 *µ*g/flask of pLVX-IRES-Puro-RFP-GFP-fis1 plasmid, 1.5 *µ*g/flask of pPAX2 plasmid, and 0.5 *µ*g/flask of pMD2.G plasmid were transfected into the HEK293T cells with TurboFect™ Transfection Reagent (Thermo Fisher, R0533) in accordance with the manufacturer's protocol. At 48hr posttransfection, the cells were centrifuged at 12,000 *g* for 5 min, and the resulting supernatant containing the packaged lentivirus was collected, aliquoted, and stored at−80°C.

### 2.11. Detection of Autophagy by Confocal Laser Microscopy

NIH-3T3 cells were seeded at a density of 6 × 10^4^ cells/well into a 24-well plate with glass cell slides preinstalled in the wells, and then placed in a cell culture incubator for 12 hr. The pGFP-LC3-RFP plasmid was transfected into these NIH-3T3 cells with TurboFect™ Transfection Reagent (Thermo Fisher, R0533) in accordance with the manufacturer's protocol. At 24hr posttransfection, the complete cell medium containing 3-*µ*M gossypol was replaced, and the cells were further cultured for 24 hr. The cells were finally fixed and sealed, and the appearance of autophagosomes in the cells was observed and photographed under a Zeiss LSM710 confocal microscope.

### 2.12. Determination of Virus Titer

Virus titers were determined by the 50% tissue culture infective dose (TCID_50_) assay. PK-15 cells were seeded into 96-well plates (1 × 10^4^ cells/well), cultured overnight, and then infected with virus fluid samples that had been serially diluted (10^−1^−10^−11^). Each dilution was tested in eight replicate wells, and cells in the last column of the 96-well plate were left uninoculated as a control. After the plates were incubated at 37°C for 1 hr for viral adsorption, the adsorption solution was discarded, the cells were washed three times with PBS, and 200 *µ*L of maintenance solution was added into each well. The cells were further cultured for 3–5 days to observe the cytopathic change, and then the TCID_50_ value was calculated by using the Reed–Muench method.

### 2.13. Detection of Intracellular ATP

After PK-15 and NIH-3T3 cells had been treated with gossypol or DMSO at different doses for the indicated period of time, the intracellular ATP of each sample was measured with the Enhanced ATP Assay kit (S0027, Beyotime), used in accordance with the operating procedures specified in the manufacturer's instructions.

### 2.14. Atomic Force Microscopy (AFM)

AFM analysis was conducted with an MFP3D Infinity-Asylum Research AFM in tapping mode (Oxford Instruments PLC). In brief, PK-15 cells were seeded at a cell density of 2 × 10^4^ cells/well in 24-well plates with cell slides preset into the wells. After being cultured for 12 hr, they were precooled at 4°C for 1 hr, the original medium was discarded, and the cells were washed three times with precooled PBS. They were then incubated with precooled PRV HN1201 virus solution (MOI = 1,000) containing gossypol (final concentration 3 *µ*M) or DMSO at 4°C for 1 hr (for adsorption). Cell culture medium-treated wells containing DMSO were included as blank cell controls. At end of the adsorption period, the cells were washed three times with precooled PBS, and the plate was opened and placed in a biological safety cabinet overnight until dried by air. The cell slides were then removed, fixed face up onto a glass slide, and tested under an atomic force microscope.

### 2.15. Molecular Docking Analysis

The crystal structure of gB protein was downloaded from the PDB database for use in a docking analysis. The PDB ID was 6ESC, and the 3D structure of the small molecule gossypol was downloaded from the PUBCHEM database and subjected to energy minimization under an MMFF94 force field. The molecular docking analysis was performed by using AutoDock Vina 1.1.2 software, and all receptor proteins, including the removal of water molecules, salt ions, and small molecules, were treated with PyMol 2.5 before docking. The docking box was then set to wrap the entire protein structure. The well-treated small molecule gossypol and gB protein were converted into the PDBQT format required for docking by using the AutoDock Vina 1.1.2 by ADFRsuite 1.0. In the docking, the exhaustiveness of the global search was set to 32, and the rest of the parameters remained set at the default settings. The highest scoring docking conformation was considered to be the binding conformation, and the docking results were finally visualized by using PyMol 2.5 software.

### 2.16. Data Statistics and Analysis

The presented experimental data were obtained from at least three independent repeated tests. Data processing was performed by using Graphpad Prism 8, Microsoft Excel, ImageJ, and other software. The results are expressed in this manuscript as the mean ± standard deviation (mean ± SD). All statistical analyses were performed using a *t*-test or one-way ANOVA. Differences with a *P*-value of <0.05 were considered significant.

### 2.17. Generation of Atg5 Knockout Cell Line PK-15 Atg5^KO^ Using CRISPR-Cas9

The Atg5 knockout cell line PK-15 Atg5^KO^ was generated according to a previous study [22]. In brief, sgRNAs targeting porcine ATG5 were synthesized and cloned into the lentiCRISPR v2 vector (Addgene, 52961). HEK293T cells were seeded in 10-cm dishes at 4 × 10^6^ per dish. The next day, the cells were cotransfected with 1.5-*μ*g psPAX2 (Addgene, 12260), 0.5-*μ*g pMD2.G (Addgene, 12259), and 2-*μ*g lentiCRISPR v2 (with indicated sgRNA) using Lipofectamine 3000 (Invitrogen, L3000015). At 48 hr posttransfection, culture medium containing lentivirus was collected and used for infection of PK-15 cells followed by selection in culture medium containing puromycin (4 *μ*g/mL; Solarbio, P8230) for another 7 days. Single clonal Atg5 knockout cells were obtained by serial dilution and verified by Sanger sequencing and western blot. The sequence of sgATG5 is 5′- CACCGAGAAGACATTAGTGAGATATGG-3′.

## 3. Results

### 3.1. Gossypol Inhibited the Proliferation of PRV In Vitro

**Figure 1:**
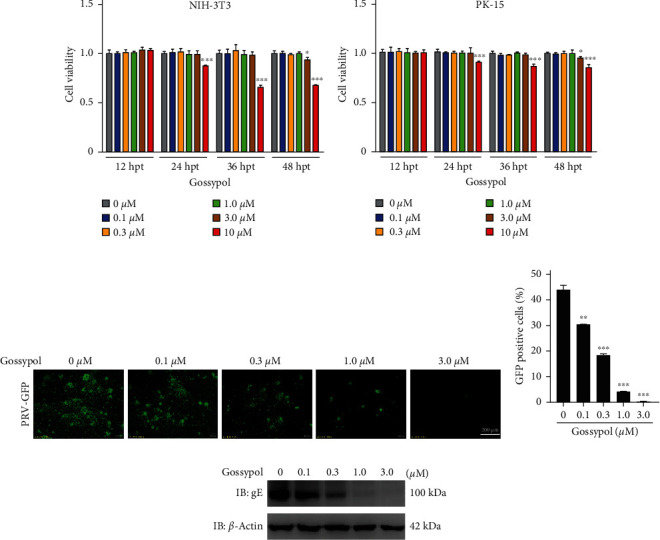
The anti-PRV effect of gossypol. (a, b) The cell viability of NIH-3T3 (a) and PK-15 (b) cells treated with gossypol (0–10 *µ*M) for 12, 24, 36, or 48 hr, as detected by CCK-8.  ^*∗*^*P*  < 0.05 and  ^*∗∗∗*^*P*  < 0.001. (c) PK-15 cells were pretreated with the indicated concentrations of gossypol for 4 hr and then inoculated with gossypol-containing rPRV HN1201-EGFP-Luc (MOI = 0.01) virus solution at 37°C for 1 hr. The infecting inoculum was replaced with gossypol-containing complete medium for 24 hr, and the cell fluorescence was detected under a fluorescence microscope. Scale bar, 200 *µ*m. (d) EGFP-positive PK-15 cells were detected by flow cytometry (cells were treated as described above in part (c)).  ^*∗∗*^*P*  < 0.01 and  ^*∗∗∗*^*P*  < 0.001. (e) Western blot analysis of PK-15 cells that were treated described above as in part (c) but infected with PRV HN1201 (MOI = 1), conducted with the indicated antibodies.

To determine the safe-use concentration of gossypol in cells, the cytotoxicity of gossypol to NIH-3T3 and PK-15 cells was determined by using a CCK-8 kit. Gossypol had no obvious toxicity to NIH-3T3 cells and PK-15 cells within 36 hr after treatment within the effective anti-PRV concentration range of 3 *µ*M (Figures 1(a) and 1(b)). To conduct a comprehensive evaluation of the effect of gossypol on PRV proliferation, PK-15 cells were treated with gossypol and then infected with recombinant (r) PRV HN1201-EGFP-Luc or PRV HN1201. The results of flow cytometry (Figure 1(c)), fluorescence microscopy (Figure 1(d)), and western blot (Figure 1(e)) demonstrated that gossypol significantly inhibited the proliferation of PRV in a dose-dependent manner. In NIH-3T3 cells, gossypol also had a significant inhibitory effect on PRV (*Supplementary 1*).

### 3.2. Gossypol Did Not Exert Its Anti-PRV Effects through mtDNA Leakage and Stimulation of IFN-*β* Production

**Figure 2:**
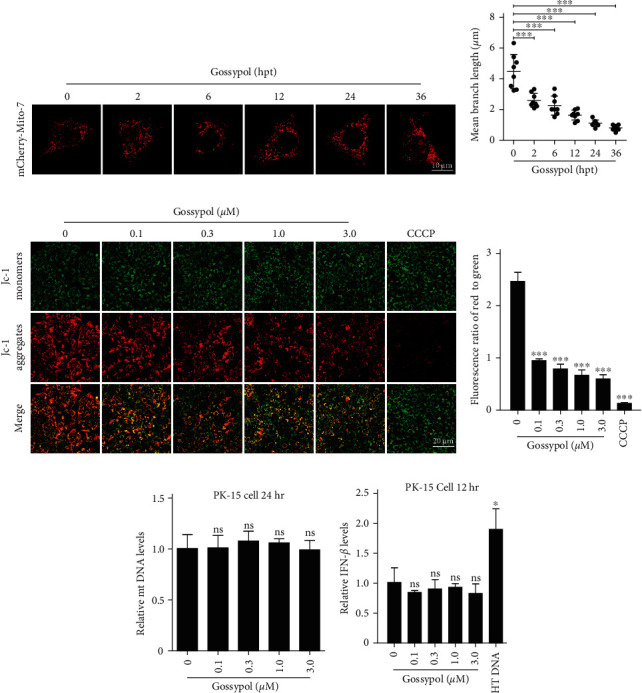
Anti-PRV activity of gossypol was independent of mtDNA leakage and IFN-*β* production. (a, b) Confocal laser microscopy observation of the mitochondrial morphology (a) and a statistical analysis of the mitochondrial length at specific time points (0, 2, 6, 12, 24, and 36 hr), conducted with ImageJ software, (b) of PK-15 cells after their treatment with 3 *µ*M gossypol. Scale bar, 10 *µ*m;  ^*∗∗∗*^*P*  < 0.001. (c, d) PK-15 cells were treated with the indicated concentrations of gossypol for 24 hr, and then stained with JC-1. The change in mitochondrial membrane potential was observed by confocal laser microscopy (c), and the ratio of red and green fluorescence intensity of the images was analyzed using ImageJ software (d). Scale bar, 20 *µ*m;  ^*∗∗∗*^*P*  < 0.001. (e, f) qPCR Assay of intracellular mtDNA (e) and IFN-*β* (f) expression change in PK-15 cells that were treated with the indicated concentrations of gossypol (0, 0.1, 0.3, 1, or 3 *µ*M) for 24 hr. NS, not significant,  ^*∗*^*P*  < 0.05.

Some of the pharmacological properties of gossypol, such as its anti-cancer and anti-inflammatory properties, depend on its interaction with cellular mitochondria [15, 23–25]. Mitochondria are important organelles in eukaryotic cells because they are not only energy factories and key metabolic hubs in cells [17], but also perform important functions in the innate immune response process [18]. For these reasons, the effect of gossypol (within the range of effective anti-PRV concentrations) on the mitochondria was investigated in PK-15 cells. The results showed that, following gossypol treatment, the morphology of mitochondria in cells gradually changed from branched to having a punctate shape (Figure 2(a)). A statistical analysis of the mitochondrial length in gossypol-treated and untreated cells performed with ImageJ software revealed that the mitochondrial length in cells gradually shortened over time in response to gossypol treatment (Figure 2(b)), indicating that gossypol can lead to changes in mitochondrial morphology in PK-15 cells.

Determining whether the mitochondrial membrane potential is normal and crucial for a comprehensive assessment of the mitochondrial functional status [26]. To further evaluate the effect of gossypol on the functional status of mitochondria, the effect of gossypol at different concentrations on the mitochondrial membrane potential was monitored by using JC-1 probes. As the concentration of gossypol increased, the intracellular red fluorescence gradually decreased, and the green fluorescence increased slightly (Figure 2(c)). The statistical analysis performed with ImageJ software showed that the ratio of red to green fluorescence decreased gradually with increases of the gossypol concentration (Figure 2(d)), indicating that gossypol reduced the mitochondrial membrane potential in a dose-dependent manner in PK-15 cells.

Because the results described above indicate that gossypol has an effect on the mitochondria of PK-15 cells, we speculated that gossypol might inhibit the proliferation of PRV by causing mitochondrial damage, mtDNA leakage, and stimulating cytoplasmic DNA receptors to increase the IFN-*β* expression level. To test this possibility, PK-15 cells were treated with complete cell culture medium containing different concentrations of gossypol for 24 hr, after which the total cellular DNA and RNA were extracted, and the levels of mtDNA and IFN-*β* in the cells were measured by qPCR. The results showed that treatment with gossypol for 24 hr at concentrations of ≤3 *µ*M did not induce a decrease in the level of mitochondrial DNA in PK-15 cells (Figure 2(e)), nor did it stimulate an increase in the intracellular IFN-*β* expression level (Figure 2(f)), indicating that gossypol did not exert its anti-PRV effect through mtDNA leakage and IFN-*β* production.

### 3.3. The Anti-PRV Effect of Gossypol in Cells Is Independent of ROS

**Figure 3:**
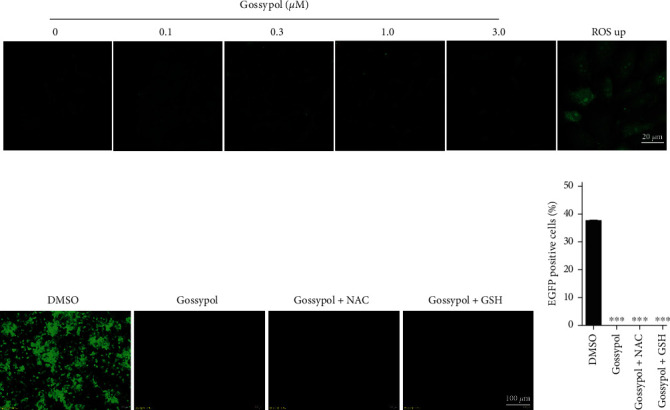
Gossypol did not exert anti-PRV effects by stimulating ROS. (a) The level of ROS in PK-15 cells that were treated with the indicated concentrations of gossypol for 24 hr was detected with a Reactive Oxygen Species Assay kit; the cells were observed and photographed under a confocal laser microscope. Scale bar, 20 *µ*m. (b) PK-15 cells were treated with DMSO, gossypol (3 *µ*M), gossypol (3 *µ*M) +NAC (0.1 mg/mL), or gossypol (3 *µ*M) +GSH (1 mg/mL) and then inoculated with rPRV HN1201-EGFP-Luc (MOI = 0.01). The viral proliferation after 24 hr was observed under a fluorescence microscope. Scale bar, 100 *µ*m. (c) Cells were treated and inoculated as described above in part (b), and the proportion of EGFP-positive cells was determined by flow cytometry  ^*∗∗∗*^*P*  < 0.001.

ROS generated during intracellular mitochondrial oxidative phosphorylation plays an important role in immune responses, and high levels of ROS generated under some stimulating factors even have direct killing effects on pathogens [27, 28]. Studies have shown that gossypol can cause increased ROS levels in some cells [29–31]. To investigate whether the inhibitory effect of gossypol on PRV in PK-15 cells depends on an increase in ROS levels, the ROS levels in PK-15 cells treated with different concentrations of gossypol for 24 hr were measured with a ROS Assay Kit. There was no obvious visible fluorescence in PK-15 cells treated for 24 hr with gossypol at a concentration of less than 3 *µ*M, whereas obvious intracellular green fluorescence was observed in the positive control (ROS up-treated cells) (Figure 3(a)), indicating that the treatment of PK-15 cells for 24 hr with gossypol at a concentration of less than 3 *µ*M did not cause increased levels of intracellular ROS.

To further investigate whether the inhibitory effect of gossypol on PRV in PK-15 cells was dependent on increased ROS levels, when PK-15 cells were treated with gossypol (3 *µ*M), the antioxidant N-acetyl-L-cysteine (NAC) or reduced glutathione (GSH) were also added to the cells, and the ability of these antioxidants to alleviate the inhibitory effect of gossypol on rPRV HN1201-EGFP-Luc was determined by conducting fluorescence microscopy and flow cytometry. The inhibitory effect of gossypol on PRV was not relieved at all by the addition of the antioxidant NAC or GSH, which further demonstrated that the anti-PRV effect of gossypol is independent of ROS in these cells (Figures 3(b) and 3(c)).

### 3.4. The Inhibitory Effect of Gossypol on PRV Is Independent of Its Induced Decrease in Intracellular ATP Levels

**Figure 4:**
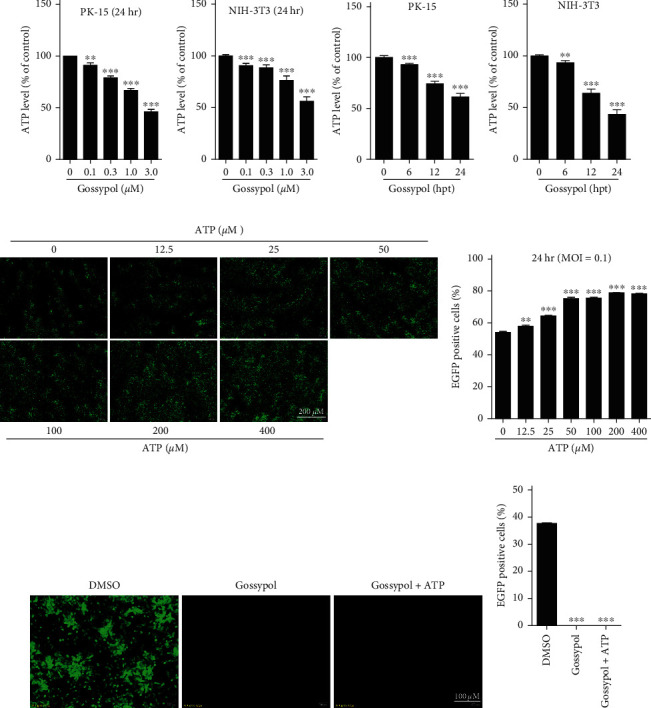
Anti-PRV effect of gossypol was ATP-independent. (a, b) ATP detection after the treatment of PK-15 (a) and NIH-3T3 (b) cells with the indicated concentrations of gossypol (0, 0.1, 0.3, 1, or 3 *µ*M) for 24 hr.  ^*∗∗*^*P*  < 0.01 and  ^*∗∗∗*^*P*  < 0.001. (c, d) ATP detection at different lengths of time (0, 6, 12, or 24 hr) after the treatment of PK-15 (c) and NIH-3T3 (d) cells with 3 *µ*M gossypol.  ^*∗∗*^*P*  < 0.01 and  ^*∗∗∗*^*P*  < 0.001. (e) PK-15 cells were treated with the indicated concentrations of ATP and inoculated with rPRV HN1201-EGFP-Luc (MOI = 0.1). The viral proliferation after 24 hr was observed under a fluorescence microscope. Scale bar, 200 *µ*m. (f) Cells were treated and inoculated as described above in part (e), and the proportion of EGFP-positive cells after 24 hr was determined by flow cytometry.  ^*∗∗*^*P*  < 0.01,  ^*∗∗∗*^*P*  < 0.001. (g) PK-15 cells were treated with DMSO, gossypol (3 *µ*M), or gossypol (3 *µ*M) +ATP (100 *µ*M) and then inoculated with rPRV HN1201-EGFP-Luc (MOI = 0.01). The cells were observed and photographed 24 hr later under a fluorescence microscope. Scale bar, 100 *µ*m. (h) Cells were treated and inoculated as described above in part (g), and the proportion of EGFP-positive cells was determined by flow cytometry.  ^*∗∗∗*^*P*  < 0.001.

Viruses utilize the synthesis pathways and organelles of host cells to promote their replication, and intracellular ATP provides the necessary energy for this process. When ATP was experimentally depleted, the DNA packaging and viral capsid maturation of herpes simplex virus (HSV) were blocked [32]. Here, the effect of gossypol on the intracellular ATP levels was investigated. The results showed that at 24 hr after the treatment of PK-15 or NIH-3T3 cells with gossypol, the intracellular ATP level decreased in a dose-dependent manner (Figures 4(a) and 4(b)). When PK-15 and NIH-3T3 cells were treated with 3-*µ*M gossypol and the changes in intracellular ATP levels were monitored at different time points after treatment, the intracellular ATP levels were found to gradually decrease over time (Figures 4(c) and 4(d)). Thus, gossypol caused a decrease in the intracellular ATP levels of PK-15 and NIH-3T3 cells in both a time- and dose-dependent manner.

Before investigating whether the inhibitory effect of gossypol on PRV is dependent on ATP, the effect of ATP on PRV proliferation was first determined by adding ATP alone to PK-15 cells. As the ATP concentration increased, the intracellular fluorescence level also gradually increased, indicating that ATP can promote the proliferation of PRV (Figures 4(e) and 4(f)). However, when PK-15 cells were simultaneously treated with gossypol and ATP before being inoculated with rPRV HN1201-EGFP-Luc, the addition of ATP had no obvious influence on the inhibitory effect of gossypol on PRV (Figures 4(g) and 4(h)), indicating that the anti-PRV effect of gossypol is ATP-independent.

### 3.5. Gossypol Inhibited PRV Independently of Its Induction of Autophagy

**Figure 5:**
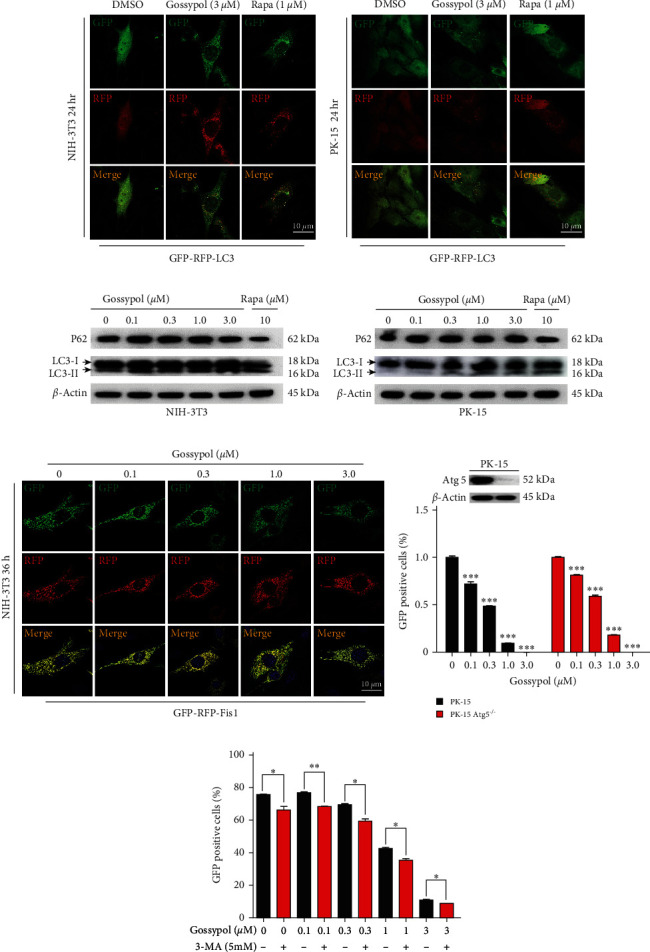
Anti-PRV effect of gossypol was independent of the autophagy it induced. (a, b) NIH-3T3 (a) and PK-15 (b) cells were transfected with the GFP-RFP-LC3 plasmid and then treated with DMSO, gossypol (3 *µ*M), or Rapa (1 *µ*M) for 24 hr. The GFP and RFP fluorescence levels were detected under a confocal microscope. Scale bar, 10 *µ*m. (c, d) NIH-3T3 (c) and PK-15 (d) cells were treated with gossypol (0, 0.1, 0.3, 1, or 3 *µ*M) or Rapa (10 *µ*M) for 24 hr, and then the expression of LC3-I, LC3-II, and P62 proteins was detected by western blot. (e) Mitophagy was detected after NIH-3T3 cells were treated with gossypol. NIH-3T3 cells were infected with pLVX-IRES-Puro-RFP-GFP-fis1 lentivirus, which has a mitophagy-indicating function, and 12 hr later, the cells were treated with complete medium containing the indicated concentration of gossypol (0, 0.1, 0.3, 1, or 3 *µ*M) for 36 hr. The cells were fixed and sealed, and the occurrence of intracellular mitophagy was observed and photographed under a confocal microscope. Scale bar, 10 *µ*m. (f) (Top) Detection of Atg5 in PK-15 Atg5^WT^ and PK-15 Atg5^KO^ cells by western blot; (Bottom) Atg5^WT^ and Atg5^KO^ PK-15 cells were treated with the indicated concentration of gossypol (0, 0.1, 0.3, 1, or 3 *µ*M) and then infected with rPRV HN1201-EGFP-Luc (MOI = 0.01) for 24 hr. The proportion of EGFP-positive cells was determined by flow cytometry, and the results were normalized to those of the control group. (g) PK-15 cells were treated with the indicated concentration of gossypol (0, 0.1, 0.3, 1, or 3 *µ*M) alone or cotreated with the indicated concentration of gossypol (0, 0.1, 0.3, 1, or 3 *µ*M) and 3-MA (5 mM) and then infected with rPRV HN1201-EGFP-Luc (MOI = 0.01) for 36 hr. The proportion of EGFP-positive cells was determined by flow cytometry  ^*∗*^*P*  < 0.05,  ^*∗∗*^*P*  < 0.01, and  ^*∗∗∗*^*P*  < 0.001.

It has been reported that gossypol can induce autophagy in certain cell types [33–36], and some studies showed that the occurrence of intracellular autophagy can inhibit the proliferation of PRV [22, 37]. To investigate whether gossypol inhibits PRV by inducing autophagy in cells, as described in the previous studies [38], the GFP-RFP-LC3 dual fluorescent indicator plasmid was used to detect autophagy in NIH-3T3 and PK-15 cells after their treatment with gossypol. In these experiments, a positive control of 1-*µ*M rapamycin (Rapa) and a negative control of DMSO were included. Confocal microscopy revealed that the number of spots with yellow and red fluorescence was increased in both gossypol- and Rapa-treated cells, indicating that gossypol triggered autophagy and enhanced autophagic flux (Figures 5(a) and 5(b)). Western blot results showed that, with increasing concentrations of gossypol in PK-15 or NIH-3T3 cells, the levels of autophagy-selective substrate P62 protein gradually decreased, while those of the autophagosome formation marker LC3-II protein gradually increased (Figures 5(c) and 5(d)), indicating that gossypol activated autophagy.

We further investigated whether gossypol could induce mitophagy in NIH-3T3 cells by using packaged lentivirus pLVX-IRES-Puro-RFP-GFP-fis1 (RFP and GFP dual fluorescent mitochondrial labeling system). With increasing gossypol concentrations, the mitochondrial morphology in the cells became gradually fragmented, but after the merging of RFP and GFP, all the mitochondria appeared as bright yellow (Figure 5(e)). Notably, if mitophagy had occurred, the mitochondria would have appeared red after double fluorescence merging; thus, these results indicated that gossypol did not induce mitophagy in NIH-3T3 cells under the experimental conditions.

To explore whether gossypol exerted its anti-PRV effect by inducing autophagy in cells, the PK-15 cell line Atg5^KO^, which has the key autophagy gene *Atg5* knocked out, and the autophagy inhibitor 3-Methyladenine (3-MA) were used for analyzing whether an inhibition of autophagy could alleviate the anti-PRV effect of gossypol. The results showed that gossypol could effectively inhibit the proliferation of PRV in the PK-15 Atg5^KO^ cell line despite the knockdown of *Atg5* for inhibition of autophagy in these cells (Figure 5(f)). In addition, adding the autophagy inhibitor 3-MA to inhibit autophagy did not alleviate the anti-PRV effect of gossypol, but instead even further inhibited the proliferation of PRV (Figure 5(g)). Although the mechanism is not clear, the inhibitory effect of gossypol on PRV is autophagy-independent.

### 3.6. Gossypol Exerted Its Anti-PRV Effect by Inhibiting PRV Adsorption on the Cell Surface

**Figure 6:**
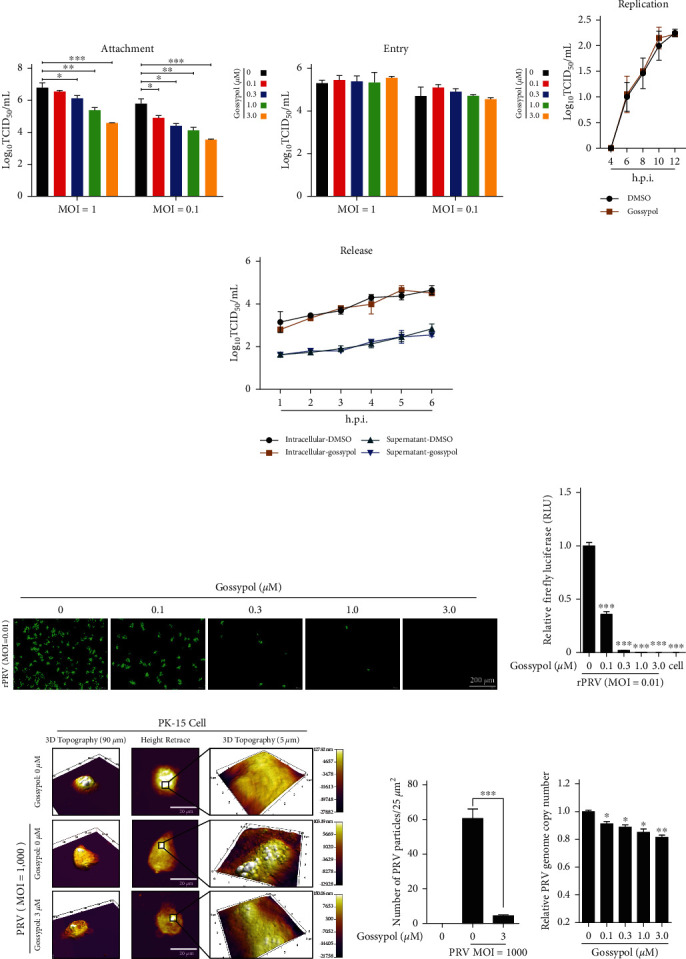
Gossypol exerted an antiviral effect by inhibiting PRV adsorption on the cell surface. (a) Antiviral effects of gossypol in PRV adsorption. PK-15 cells were precooled for 1 hr at 4°C, then incubated at 4°C for 1 hr with PRV HN1201 (MOI = 0.1 or MOI = 1) that had been precooled at 4°C, accompanied by different concentrations of gossypol (0, 0.1, 0.3, 1, or 3 *µ*M). After the adsorption solution was discarded, the cells were washed three times with PBS and then cultured in maintenance solution at 37°C for 24 hr. The cells were frozen and thawed three times to collect the virus, and its TCID_50_ was measured.  ^*∗*^*P*  < 0.05,  ^*∗∗*^*P*  < 0.01, and  ^*∗∗∗*^*P*  < 0.001. (b) Antiviral effects of gossypol in PRV entry. PRV HN1201 (MOI = 0.1 or MOI = 1) was adsorbed onto PK-15 cells for 1 hr at 4°C. After the adsorption solution was discarded, the cells were washed three times with precooled PBS, and incubated at 37°C for 2 hr in maintenance solution containing the indicated concentration of gossypol (0.1, 0.3, 1, or 3 *µ*M) or DMSO. The cells were then washed three times with PBS at room temperature, and further cultured in maintenance solution for 24 hr. The cells were frozen and thawed three times to collect the virus, and its TCID_50_ was measured. (c) Antiviral effects of gossypol in PRV replication. PRV HN1201 (MOI = 1) was adsorbed onto PK-15 cells for 1 hr at 4°C. The cells were washed three times with PBS at room temperature and then cultured at 37°C for 2 hr in maintenance solution. The maintenance solution was replaced with fresh maintenance solution containing DMSO or 3 *µ*M gossypol, and samples were collected at 4, 6, 8, 10, and 12 hr after the addition of gossypol. The TCID_50_ was measured after the cells were frozen and thawed three times. (d) Antiviral effects of gossypol in PRV release. PRV HN1201 (MOI = 1) was adsorbed onto PK-15 cells for 1 hr at 4°C. The cells were then cultured at 37°C in maintenance solution for 12 hr, washed three times with PBS, and cultured in maintenance solution containing the indicated concentrations of gossypol (0.1, 0.3, 1, or 3 *µ*M) or DMSO. The supernatants and cells were collected at different lengths of time (1, 2, 3, 4, 5, or 6 hr) after the addition of gossypol, and the TCID_50_ of the virus was measured after the cells were frozen and thawed three times. (e, f) Inhibitory effect of different concentrations of gossypol during adsorption of PRV virus. PK-15 cells were inoculated with recombinant virus rPRV HN1201-EGFP-Luc (MOI = 0.01) in the presence of the indicated concentration of gossypol (0, 0.1, 0.3, 1, or 3 *µ*M) at 4°C for 1 hr to allow adsorption. At 24 hr after inoculation, the effect of gossypol on the PRV adsorption was determined by fluorescence microscopy (e) and firefly luciferase activity assay (f)  ^*∗∗∗*^*P*  < 0.001. (g, h) Detection of virus particle uptake on cell surfaces by atomic force microscopy. PRV HN1201 (MOI = 1,000) was adsorbed onto PK-15 cells in the presence of DMSO or 3 *µ*M gossypol for 1 hr at 4°C. DMSO-treated noninoculated cells were included as a control. After the cells were washed three times with precooled PBS, the cell slides were removed and dried, then fixed face up on a glass slide, and the absorption of virus particles on the cell surface was detected under an atomic force microscope (g). The statistics of PRV particles in multiple fields of view was conducted (h)  ^*∗∗∗*^*P*  < 0.001. (i) Virus attachment in the presence of different concentrations of gossypol (0, 0.1, 0.3, 1, or 3 *µ*M). PRV HN1201 (MOI = 10) was adsorbed onto PK-15 cells in the absence or presence of gossypol (0, 0.1, 0.3, 1, or 3 *µ*M) for 1 hr at 4°C. After the cells were washed three times with ice-cold PBS, the total DNA was extracted, PRV gH DNA was detected by qPCR, and virus attachment was detected  ^*∗*^*P*  < 0.05, and  ^*∗∗*^*P*  < 0.01.

To explore whether gossypol has a direct inactivation effect on PRV, the PRV HN1201 virus was pretreated with different concentrations of gossypol at 37°C for 1 hr, then the excess compound was removed by ultracentrifugation and the virus particles were purified, and the infectivity of the purified virions was determined via the titer determination method. Compared with the control group (which was not treated with gossypol), the group treated with a concentration of ≤3 *µ*M of gossypol had no direct inactivation of PRV in vitro (*Supplementary 2*). To investigate the mechanism by which gossypol inhibits PRV, the effect of gossypol on the PRV life cycle was examined by the virus titer method. Regardless of the inoculation dose (MOI = 0.1 or MOI = 1), with increasing concentrations of gossypol applied in the virus adsorption stage, the PRV titer gradually decreased (Figure 6(a)); however, gossypol had no obvious effect on the viral entry, replication, and release stages of PRV-infected cells (Figure 6(b)–6(d)), indicating that gossypol specifically inhibited the virus adsorption stage in PRV-infected cells.

The effect of gossypol on the virus adsorption stage was then investigated more closely. In the experiment, rPRV HN1201-EGFP-Luc (MOI = 0.01) was added at the adsorption stage at 4°C, the adsorbed virus solution containing gossypol was discarded, and the cells were further cultured for 24 hr in a maintenance solution before the fluorescence of the cells was observed with a fluorescence microscope and the firefly luciferase activity was detected after cell lysis. During the virus adsorption stage, as the concentration of gossypol increased, the amount of fluorescence in the cells gradually decreased (Figure 6(e)). When the gossypol concentration was 3 *µ*M, the proliferation of the virus was almost completely inhibited. The firefly luciferase activity detection results showed that gossypol inhibited the adsorption of PRV, which was consistent with our fluorescence microscopy observations (Figure 6(f)), indicating that gossypol exerted its anti-PRV effect by inhibiting the adsorption of virus on the cell surface.

To directly observe the effect of gossypol on PRV adsorption, AFM was performed to measure the number of viruses adsorbed on the cell membrane in cells treated with varying doses of gossypol. When gossypol was not added, more virus adsorption was observed on the cell surface, whereas when 3-*µ*M gossypol was added, the number of adsorbed viruses on the cell surface was significantly lower (Figures 6(g) and 6(h)), which additionally confirmed the inhibitory effect of gossypol on the adsorption stage of PRV.

The effect of gossypol on the adsorption stage of PRV was further examined by qPCR experiments. PRV HN1201 (MOI = 10) virus adsorption was carried out in the presence of various amounts of gossypol (final concentrations: 0, 0.1, 0.3, 1, or 3 *µ*M). After adsorption at 4°C for 1 hr, the cells were collected, and total DNA was extracted for use in a qPCR assay of the PRV genome. The results show that gossypol had a significant inhibitory effect on the adsorption of PRV (Figure 6(i)).

### 3.7. Molecular Docking Analysis Showed Binding of Gossypol to PRV gB Trimers

**Figure 7:**
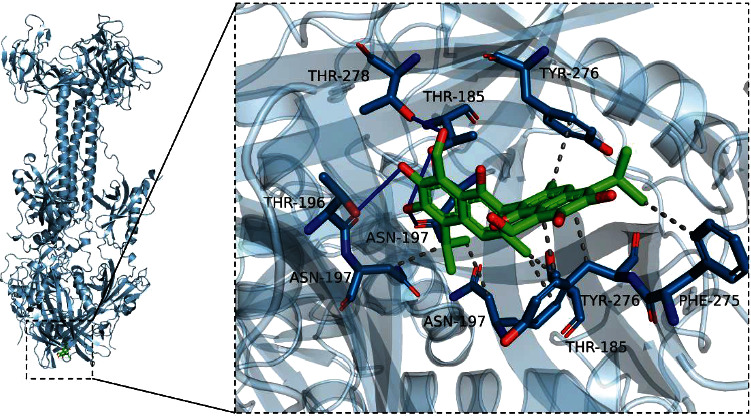
Binding of gossypol to PRV gB trimer. Overall (left) and partial (right) views of the binding mode for gossypol and gB trimer obtained from a molecular docking analysis. The cyan stick represents the compound gossypol, the light blue cartoon represents the gB protein, the blue line represents hydrogen bonding, and the gray dotted line represents a hydrophobic interaction.

The conserved gB in PRV is the main protein responsible for driving the fusion of the viral envelope with the host cell membrane and binding to the cell membrane in a cholesterol-dependent manner through its fusion loops. The binding of gossypol to the PRV gB protein trimer was predicted by using molecular docking simulation technology, and a docking study of gossypol and PRV gB protein trimer was performed using Vina 1.1.2 software. The predicted structure is shown in Figure 7. Gossypol binds to the fusion loop region at the bottom of the trimer; it can form a hydrogen-bond with THR-185, THR-196, ASN-197, and THR-278, and it can undergo hydrophobic interactions with THR-185, ASN-197, PHE-275, and TYR-276. A negative binding affinity number indicates the possibility of binding, and generally compounds with a binding affinity value of less than −6 kcal/mol are considered to be more likely to bind. For the gossypol–PRV gB protein trimer complex, the docking software produced a binding affinity score of −8.8 kcal/mol, suggesting that gossypol binds well to the trimeric gB.

## 4. Discussion

This study found that gossypol has a notable inhibitory effect on PRV. It has been reported that some pharmacological properties of gossypol, such as its anti-cancer and anti-inflammatory properties, depend on its interaction with cellular mitochondria [15, 23]. In the present study, we observed that gossypol could cause the mitochondrial morphology of PK-15 and NIH-3T3 cells to change from being cladodromous to being punctiform and shorten the length of the mitochondria in these cells. Additionally, we found that gossypol caused a decrease in the membrane potential of PK-15 cells. Together, these results indicated that gossypol affects the mitochondria in PK-15 and NIH-3T3 cells. Therefore, we designed experiments to test whether gossypol could inhibit the proliferation of PRV by causing mitochondrial damage, mtDNA leakage, and stimulating cytoplasmic DNA receptors to increase the expression of IFN-*β*. However, the results of those experiments revealed that, although gossypol at a concentration of less than 3 *µ*M had a significant inhibitory effect on PRV, it neither caused a reduction of mitochondrial DNA in PK-15 cells nor stimulated an increase in the intracellular IFN-*β* expression, indicating that gossypol does not exert its anti-PRV effects by inducing mtDNA leakage and further stimulating IFN-*β* production.

ROS generated in the course of intracellular mitochondrial oxidative phosphorylation play an important role in immune responses and, under certain stimuli, high levels of ROS even have direct killing effects on the pathogens [27, 28]. Studies have shown that gossypol can induce increased ROS levels [29, 31] in some cells. To explore whether the inhibitory effects of gossypol on PRV in PK-15 cells depend on an increase of ROS levels, the level of ROS in PK-15 cells treated with gossypol was measured with a commercial kit. However, the results showed that no increase in ROS levels was detected in PK-15 cells treated for 24 hr with gossypol at a concentration of less than 3 *µ*M. In addition, the combined use of the antioxidants (NAC or GSH) with gossypol did not relieve the inhibitory effects of gossypol on PRV, which proved that gossypol did not exert its anti-PRV effects by stimulating an increase in ROS levels.

Viruses promote their replication using the host cell synthesis mechanism, and ATP provides the energy required for this process. Studies have shown that ATP depletion can block the DNA packaging and capsid maturation [32] of HSV. In the present study, we confirmed that the addition of ATP significantly promoted PRV proliferation in cells. Mitochondria, as the “energy factories” of cells, are the main sites for the generation of ATP. On the basis of the previously observed result, gossypol had significant effects on mitochondrial morphology and membrane potential in PK-15 cells, the effect of gossypol on intracellular ATP levels was examined next. We found that PK-15 and NIH-3T3 cells were treated with gossypol for 24 hr exhibited a significant decrease in intracellular ATP levels compared with control-treated cells. However, the anti-PRV effect of gossypol was not significantly affected by the addition of ATP, which proves that the anti-PRV effect of gossypol is ATP-independent.

Gossypol can induce autophagy in some cells [33–36], and studies have shown that intracellular autophagy has an inhibitory effect on PRV [22, 37]. Here, gossypol at specific concentrations activated autophagy in PK-15 or NIH-3T3 cells, as confirmed by both LC3 fluorescent spot observation and western blot. However, when autophagy was inhibited by using the Atg5-knockout cell line PK-15 Atg5^KO^ or the autophagy inhibitor 3-MA, the PRV-inhibiting effects of gossypol were not relieved, suggesting that the PRV-inhibiting effects of gossypol are independent of its induced autophagy.

To further explore the anti-PRV mechanism of gossypol, the effects of gossypol on PRV adsorption, entry, replication, and release were investigated by adding gossypol to cells at different stages of PRV infection. The virus titer detection results show that gossypol significantly inhibited the adsorption of PRV, but had no significant effect on the entry, replication, or release of the virus. The qPCR assay results also confirm the clear inhibitory effect of gossypol on the adsorption of PRV. AFM observations revealed that the presence of gossypol during the phase of virus adsorption significantly reduced the quantity of viruses adsorbed on the cell surface, which further confirms the inhibitory effect of gossypol on PRV adsorption. When increasing concentrations of gossypol were tested during the adsorption stage of rPRV HN1201-EGFP-Luc, the generation of fluorescence and firefly luciferase activity in the cells was almost completely inhibited once the concentration of gossypol reached 3 *µ*M (Figures 6(e) and 6(f)). The in vitro co-incubation of gossypol and PRV in solution confirmed that gossypol has no direct inactivation effect on PRV. Therefore, gossypol exerts its antiviral effects by inhibiting the biological processes of adsorption and fusion between PRV and the cell membrane.

Studies have shown that gossypol has antiviral activity against enveloped viruses, such as COVID-19 and human immunodeficiency virus type 1 (HIV-1). To COVID-19, gossypol inhibited them by targeting RNA dependent RNA polymerases [39]. Gossypol and its amino acid derivatives can bind to the HIV-1 gp41 protein hydrophobic pocket, thereby inhibiting HIV-1-mediated membrane fusion and subsequent viral entry [40]. For the absorption and fusion of PRV on the cell surface, gB is considered to be the true fusion protein of herpes viruses. After the activation of the fusion program between PRV and the target cell membrane, the gB protein promotes liposome binding and membrane fusion by forming a continuous hydrophobic patch on the surface of the gB trimer through the key amino acid residues Trp187, Tyr192, Phe275, and Tyr276 in its fusion loop. Among them, Phe275 is considered to be deeply inserted into the hydrocarbon core of the lipid bilayer, and others form the edge, which can Phe275 is considered to be deeply inserted into the hydrocarbon core of the lipid bilayer, while Trp187, Tyr192, and Tyr276 form the edge, which can insert into the shallower membrane interface region to catalyze the fusion process [41]. In the present study, the direct binding of gossypol to the PRV gB trimer was explored by means of a molecular docking analysis. Through this analysis, we found that gossypol could effectively bind to the gB protein trimer. Interestingly, the binding site was located exactly in the region of the bottom fusion loop (Figure 7) of the gB protein trimer, and it formed a hydrophobic interaction with Phe-275 and Tyr-276. These two residues are the key amino acid residues necessary for the gB protein to perform the fusion function. The residues Thr185, Thr196, Thr197, and Thr278, which form hydrogen bonds between gossypol and gB are in extremely close proximity to other key amino acid residues of the fusion loop. Therefore, gossypol may block the adsorption and fusion of PRV on the cell surface by reversibly binding to the fusion loop region of gB protein.

In conclusion, we showed that gossypol has a significant inhibitory effect on PRV and comprehensively analyzed the anti-PRV mechanism of gossypol by investigating many possible biological effects of gossypol from damaging mitochondria, inducing ROS, stimulating autophagy, altering energy production, and interrupting the viral life cycle. Finally, we found that gossypol exerts its anti-PRV effects by inhibiting the adsorption of virus on the cell surface. Moreover, the infection of PK-15 cells by PRV could be almost completely blocked by adding an appropriate amount of gossypol in the adsorption phase. A molecular docking analysis revealed that gossypol can bind to the fusion loop of the PRV gB trimer protein, so gossypol is very likely to prevent PRV from being adsorbed or fuzed on the cell surface by blocking the fusion loop of the PRV gB trimer protein. The deeper mechanism by which PRV adsorption on the cell surface is inhibited by gossypol needs to be further studied.

## Data Availability

The data used to support the findings of this study are included within the article.

## Supplementary Materials

Supplementary 1 The anti-PRV effect of Gossypol on NIH-3T3 cells.

Supplementary 2 Detection of direct inactivation of gossypol on PRV in vitro.
