# Supplementary material for: Gossypol Affects Viral Replication by Inhibiting Pseudorabies Virus Adsorption
Source: Transbound Emerg Dis. 2023 Nov 11;2023:9073566. doi: 10.1155/2023/9073566 (PMC12017124; doi:10.1155/2023/9073566)
Supplement: Supplementary 1 — The anti-PRV effect of Gossypol on NIH-3T3 cells. [file 9073566.f1.docx]

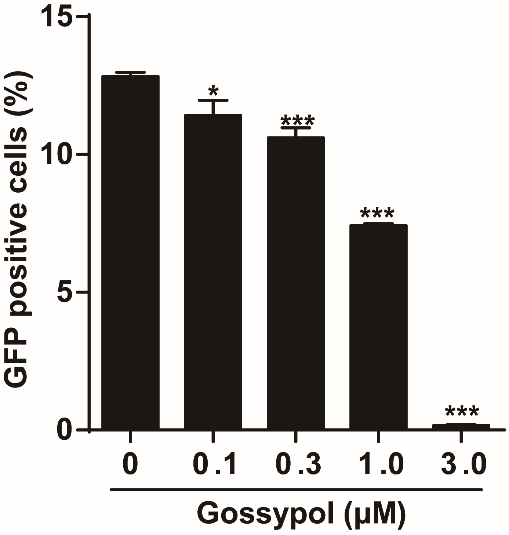


**Fig. S1** The anti-PRV effect of Gossypol on NIH-3T3 cells

NIH-3T3 cells were pretreated with the indicated concentrations of Gossypol for 4 h and then inoculated with Gossypol-containing rPRV HN1201-EGFP-Luc (MOI = 0.01) virus solution at 37 °C for 1 h. The infecting inoculum was replaced with Gossypol-containing complete medium for 24 h, and the EGFP-positive NIH-3T3 cells were detected by flow cytometry. **P* < 0.05, ****P* < 0.001.
