# Supplementary material for: Gossypol Affects Viral Replication by Inhibiting Pseudorabies Virus Adsorption
Source: Transbound Emerg Dis. 2023 Nov 11;2023:9073566. doi: 10.1155/2023/9073566 (PMC12017124; doi:10.1155/2023/9073566)
Supplement: Supplementary 2 — Detection of direct inactivation of gossypol on PRV in vitro. [file 9073566.f2.docx]

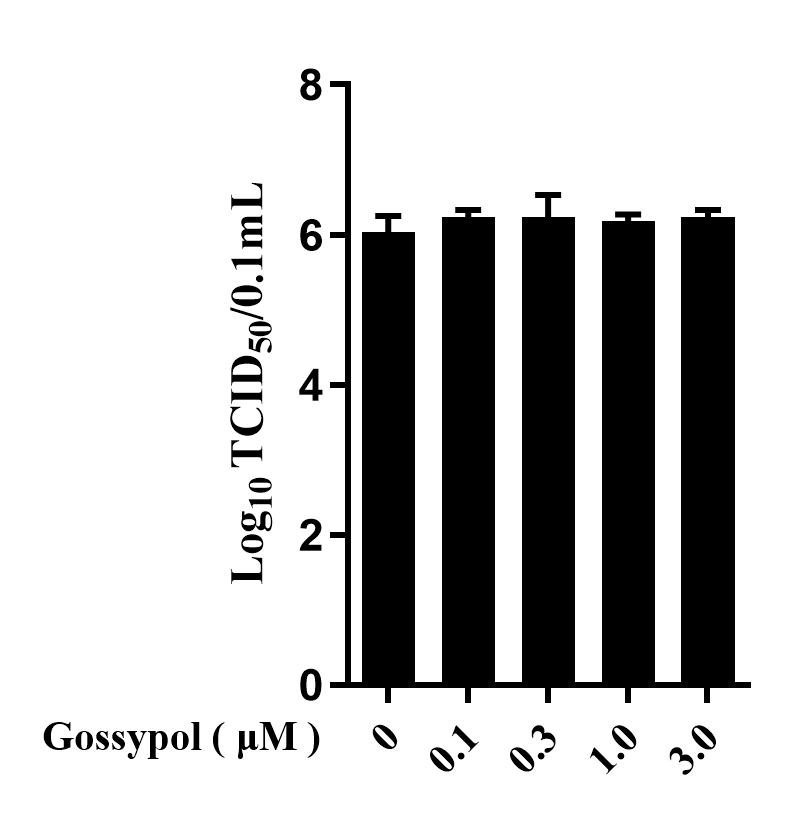


**Fig. S2** Detection of direct inactivation of gossypol on PRV in vitro

The appropriate amount of Gossypol was added to 500 μL of PRV HN1201 viral solution, resulting in final concentrations of Gossypol at 0, 0.1, 0.3, 1, and 3 μM. The mixture was then incubated at 37℃ for 1 hour, followed by high-speed centrifugation to discard the supernatant. After resuspending the virus in 500 μL of PBS, the TCID_50_ of each sample was determined.
